# A Novel Small Molecule Inhibits Hepatitis C Virus Propagation in Cell Culture

**DOI:** 10.1128/spectrum.00439-21

**Published:** 2021-07-28

**Authors:** Ahmed K. Oraby, Cassandra L. Gardner, Robert F. Needle, Hassan M. Kofahi, Kylie R. Everard, Nathan G. A. Taylor, Suzette G. Rutihinda, Jacqueline P. Barry, Kensuke Hirasawa, Paris E. Georghiou, Rodney S. Russell

**Affiliations:** a Division of BioMedical Sciences, Faculty of Medicine, Memorial University of Newfoundland, St. John’s, Newfoundland and Labrador, Canada; b Department of Pharmaceutical Organic Chemistry, Faculty of Pharmacy, Misr University for Science & Technology, Al-Motamayez District, 6th of October City, Giza, Egypt; c Department of Chemistry, Memorial University of Newfoundland, St. John’s, Newfoundland and Labrador, Canada; University of Arizona

**Keywords:** antiviral agent, virus, JFH-1, HCVcc, antiviral agents, hepatitis C virus, virus inhibition

## Abstract

Hepatitis C virus (HCV) can cause acute and chronic infection that is associated with considerable liver-related morbidity and mortality. In recent years, there has been a shift in the treatment paradigm with the discovery and approval of agents that target specific proteins vital for viral replication. We employed a cell culture-adapted strain of HCV and human hepatoma-derived cells lines to test the effects of our novel small-molecule compound (AO13) on HCV. Virus inhibition was tested by analyzing RNA replication, protein expression, and virus production in virus-infected cells treated with AO13. Treatment with AO13 inhibited virus spread in cell culture and showed a 100-fold reduction in the levels of infectious virus production. AO13 significantly reduced the level of viral RNA contained within cell culture fluids and reduced the cellular levels of HCV core protein, suggesting that the compound might act on a late step in the viral life cycle. Finally, we observed that AO13 did not affect the release of infectious virus from infected cells. Docking studies and molecular dynamics analyses suggested that AO13 might target the NS5B RNA polymerase, however, real-time RT-PCR analyses of cellular levels of HCV RNA showed only an ∼2-fold reduction in viral RNA levels in the presence of AO13. Taken together, this study revealed that AO13 showed consistent, but low-level antiviral effect against HCV, although the mechanism of action remains unclear.

**IMPORTANCE** The discovery of curative antiviral drugs for a chronic disease such as HCV infection has encouraged drug discovery in the context of other viruses for which no curative drugs currently exist. Since we currently face a novel virus that has caused a pandemic, the need for new antiviral agents is more apparent than ever. We describe here a novel compound that shows a modest antiviral effect against HCV that could serve as a lead compound for future drug development against other important viruses such as SARS-CoV-2.

## INTRODUCTION

Hepatitis C virus (HCV) is one of the leading causes of acute and chronic liver diseases and constitutes a significant health burden with an estimated 71 million people chronically infected worldwide (World Health Organization, 2017). Long-term HCV infection is often the main cause for advanced complications, including fibrosis, cirrhosis, and hepatocellular carcinoma, making HCV infection the main indicator for liver transplantation (1, 2). HCV is a positive-strand RNA virus within the *Flaviviridae* family, which also includes classical flaviviruses such as yellow fever and dengue virus. The HCV genome encodes a polyprotein that is processed into 10 different proteins: core, E1, E2, p7, NS2, NS3, NS4A, NS4B, NS5A, and NS5B (3). The nonstructural proteins NS3/4A, NS5A, and NS5B are involved in replication of the viral genome, whereas the structural proteins core, E1, and E2 are components of the virion (4, 5). There are six major genotypes (GTs) of HCV, each with multiple subtypes. Substantial regional differences exist with respect to the global distribution of HCV genotypes. GT1a and 1b are the predominant subtypes in the United States and Europe and are approximately 88% genetically similar (6, 7). Genotypes 1, 2, and 3 are most common and are responsible for more than 90% of HCV infection in North America, Europe, and Japan, while HCV GT4 is particularly prevalent in intravenous drug users in Europe and the United States (8). GT4 is mainly found in Egypt and Africa, while GT5 is found in South Africa, and GT6 in southeastern Asia (9, 10).

A combination of pegylated interferon-*α* (Peg-IFN-α) and ribavirin (RBV) was the treatment of choice for HCV infection for many years; however, this treatment had demonstrated limited efficacy and serious side effects (11, 12). Recent advances in medicinal chemistry led to the discovery of direct-acting antivirals (DAAs), which resulted in a breakthrough in the world of hepatitis C treatment. In 2011 the first DAAs were approved for treatment and the NS3/4A protease inhibitors (PIs) boceprevir and telaprevir were prescribed in combination with Peg-IFN-α and RBV to treat GT1 patients, including those with liver disease, and showed an improved sustained virologic response by up to 80% (13–15). Although the availability of protease-specific DAAs was a major advance in HCV treatment, many patients experienced serious side effects, including severe photosensitivity, so the development of better anti-HCV agents was a necessity (16). The second generation of protease inhibitors, also referred to as second-wave PIs, offered better pharmacokinetics than that of the first generation, thereby allowing for a single daily dose of these inhibitors. Although the second-wave PIs were better than the first-generation PIs, the course of treatment using these PIs should include in combination other DAAs owing to the low genetic barrier to resistance of the second-generation PIs (17).

The NS5B polymerase was the main target for antiviral development owing to its pivotal role in viral RNA synthesis. Nucleoside and nucleotide inhibitors were the first classes to target the HCV NS5B polymerase and were acting as chain terminators. Due to the conservation of the active site, these classes of inhibitors were attractive for further DAA development owing to their high inhibitory potency, pan-genotypic profile, and high generic barrier to resistance. Sofosbuvir was the first NS5B U.S. Food and Drug Administration-approved nucleoside phosphoramidate prodrug for HCV, and it is the backbone of the first oral, pan-genotypic, single-tablet regimen for the treatment of adults with genotype 1 to 6 chronic HCV infection (a combination of sofosbuvir with the NS5A inhibitor velpatasvir). However, a recent study revealed that DAAs can result in reactivation of hepatitis B infection in patients coinfected with HCV (18). Therefore, the development of novel DAAs with little to no side effects and high therapeutic indexes remains to be a priority.

The nonstructural protein NS5A is a critical protein in the HCV replication process. NS5A is an RNA binding protein that interacts with other HCV nonstructural proteins and with a variety of cellular proteins leading to potentially modulating multiple aspects of the cellular environment (19, 20). Although the development of DAAs was focused on developing inhibitors of NS3 protease and NS5B proteins, attention was directed toward NS5A since the discovery of daclatasvir, a small molecule inhibitor that exhibited picomolar EC_50_ with pan-genotypic activity (21, 22).

**FIG 1:**
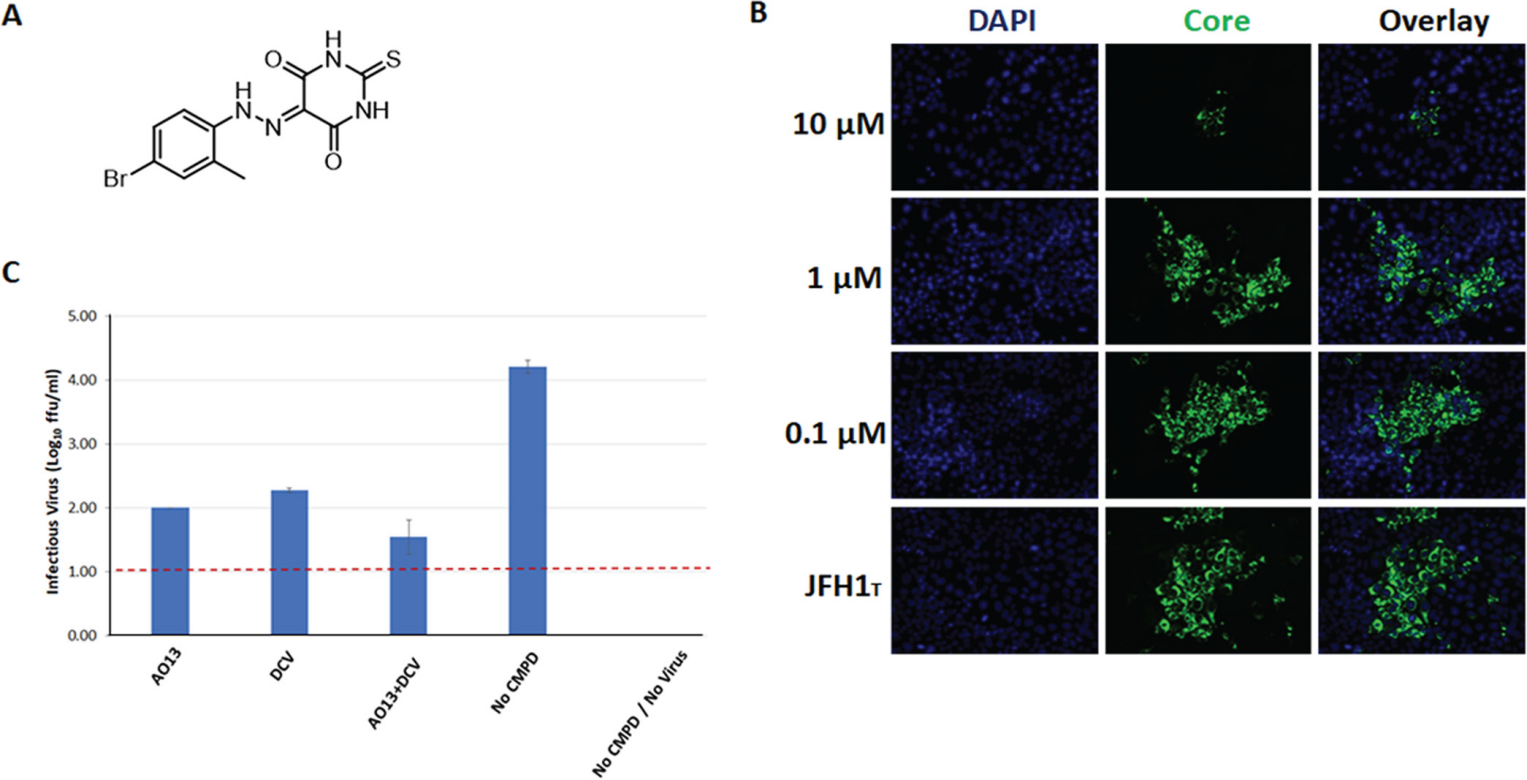
Activity of compound AO13 on HCV JFH1_T_ in cell culture. (A) Chemical structure of AO13. (B) Huh-7.5 cells were infected with JFH1_T_ at an MOI of 0.1, and compound AO13 was added at concentrations of 0.1, 1, and 10 μM. At 2 days postinfection, supernatants were collected, and Huh 7.5 cells plated in 8-well chamber slides were infected with the supernatants, and the slides were fixed and stained for HCV core for visualization. The results shown are representative of two independent experiments. (C) Effect of AO13 on extracellular virus production measured by viral titers. Infectious virus titer was measured in the presence of 10 μM AO13, 100 pM daclatasvir (DCV), and a combination of both compounds. Viral titer was measured by limiting dilution focus-forming assay performed in triplicate and is expressed as FFU/ml. The results shown are representative of two independent experiments.

In this study, we evaluated a novel heterocyclic-based small molecule, AO13 (Fig. 1) for its antiviral effect in cell culture using a GT2a cell culture-adapted strain of fully replicating HCV (JFH1_T_) (23, 24). We observed low but consistent inhibition of HCV propagation with evidence for activity at a later stage of the viral life cycle. We also employed molecular modeling to identify the putative target of the compound and potential binding mode.

## RESULTS

### Activity of AO13 on JFH1_T_ in cell culture.

The effects of AO13 on cell viability was routinely assessed by MTT assays, cell counting, and visualization of cell morphology and density upon DAPI staining and visualization by fluorescence microscopy. Concentrations of 10 μM or less showed no effects on cells, but all higher concentrations tested showed observable effects on cell viability in all methods of analysis mentioned above (data not shown). To test whether AO13 had antiviral activity against HCV, we analyzed the effects of this compound on virus spread in a nonchimeric, fully infectious HCV cell culture system. We infected Huh-7.5 cells with our cell culture-adapted strain of JFH-1 (JFH1_T_) at an MOI of 0.1 in 10-cm dishes and cultured the virus in the presence of AO13 at concentrations of 0.1, 1, and 10 μM for 2 days. To directly visualize the effects of AO13 on virus spread, supernatants from these cultures were then used to inoculate Huh-7.5 cells plated in 8-well chamber slides the previous day, again in the presence of the above concentrations of AO13. At 2 days postinfection, culture fluids were removed and the slides were fixed and stained for HCV core. We observed that compound concentrations of 0.1 and 1 μM had no effect on virus spread, whereas a concentration of 10 μM significantly inhibited the virus spread (Fig. 1B).

To quantitatively evaluate the antiviral effect of AO13 on HCV, we measured viral titers in the presence of the compound. For comparison purposes, we included daclatasvir (DCV), an NS5A inhibitor, alone and in combination with AO13. Huh-7.5 cells were infected with virus at an MOI of 0.1 in the presence of 10 μM AO13, 100 pM DCV, or a combination of both, and supernatants were harvested 48 h postinfection. The levels of virus produced in the presence of the respective compound treatments was then measured using limiting dilution focus-forming assays. The results of this experiment revealed that AO13 effected an ∼2-log reduction in infectious virus produced from infected cells (Fig. 1C). Daclatasvir also reduced virus production by ∼1.7 log, while both inhibitors together showed an almost 3-log reduction in infectious virus produced. It is important to note here that although the antiviral effects observed for AO13 and DCV were similar, we used DCV at the 50% effective concentration of 100 pM, whereas as the AO13 concentration was 10^5^-fold higher at 10 μM, indicating that AO13 is far less potent than DCV. The slight enhancement of inhibition observed when AO13 was combined with DCV was not significant enough to allow us to draw any conclusions regarding synergy or respective mechanisms of action of the two compounds. However, taking together the results of both experiments, it appears that AO13 showed potential antiviral activity against HCV.

### AO13 showed an antiviral effect in the early stages of the HCV life cycle.

**FIG 2:**
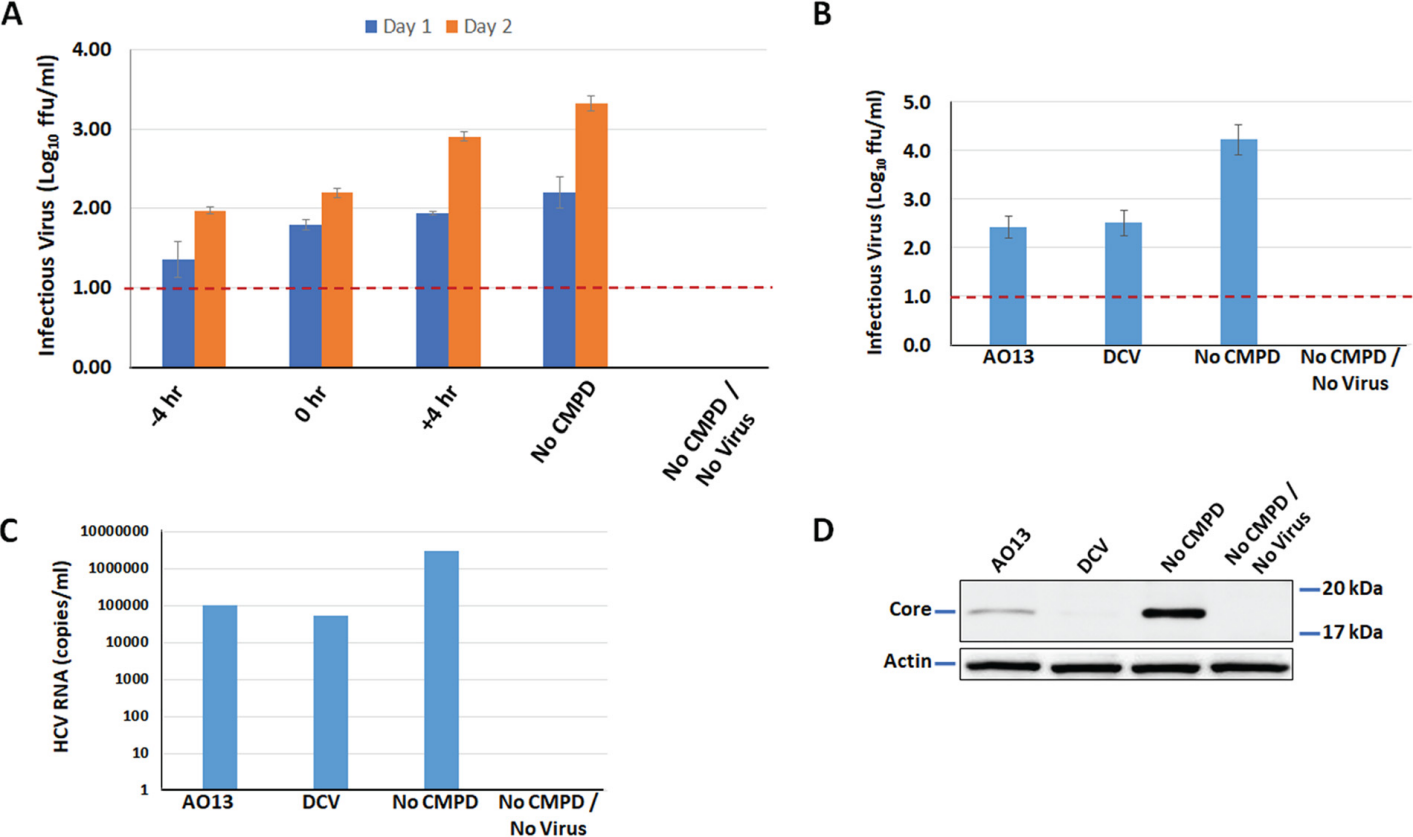
Time of addition and effects of AO13 on RNA and viral core protein expression. (A) AO13 was added at three different time points either 4 h before inoculation, at inoculation, or 4 h after virus inoculation. Extracellular culture fluids were collected on days 1 and 2 postinfection and inoculated in 8-well immunofluorescence chamber slides for infectious virus titer determination in triplicate. (B) Effect of AO13 on extracellular virus production confirmed by repeating viral titers, performed in triplicate. (C) Extracellular viral RNA levels in the presence of AO13 and DCV. (D) Effect of AO13 on intracellular HCV core expression. Concentrations of 10 μM AO13 or 100 pM DCV were used for all experiments. The results shown are representative of two independent experiments.

To investigate the possible mechanism of the compound and its potential target, we performed experiments to determine where in the viral life cycle the compound was acting. To do this, we used a time-of-addition assay in which AO13 was added at either 4 h before virus inoculation, at inoculation, or 4 h after addition of virus. Supernatants were collected on days 1 and 2 postinfection, and the virus titer was measured for each of the time points of compound addition. On both days 1 and 2, the levels of virus produced were similar to those of the no compound (No CMPD) control, but lower virus titers (∼1.3 log) were observed on day 2 when the compound was added before or at the time of inoculation (Fig. 2A). This observation suggested that AO13 acts on an early step of the viral life cycle.

### AO13 reduced viral RNA and HCV core protein expression.

To further investigate which step in the viral life cycle was affected by AO13, we examined the effects of its treatment on viral RNA replication and protein production and processing. Naive Huh 7.5 cells were infected with JFH1_T_ at an MOI 0.1 in the presence of 10 μM AO13 or 100 pM DCV as a reference drug. At 2 days postinfection, culture fluids were collected and analyzed for the levels of infectious virus and viral RNA. We found that AO13 exhibited an ∼1.8-log reduction in virus titer, similar to that of DCV (1.7-log reduction), albeit at much higher concentrations of AO13 compared to DCV (Fig. 2B). When we compared viral RNA levels in the culture fluids by real-time RT-PCR, we observed ∼1-log reductions in viral genomic RNA in the context of both AO13 and DCV treatment (Fig. 2C). These results suggest that like DCV, AO13 affects virus production through an effect on viral RNA production. To directly assess the potential effect of AO13 on viral RNA replication, we performed real-time RT-PCR on cellular HCV RNA levels, but due to the high levels of background associated with this assay, the results were inconclusive. Therefore, we next compared the levels of HCV core protein contained within the infected cells by Western blotting and found that treatment with AO13 significantly reduced the cellular levels of core protein (Fig. 2D). The effect of AO13 on HCV core expression was less than that of DCV, which showed almost no HCV core. Taken together, these results suggested that AO13 is acting on HCV RNA replication to cause a reduction in subsequent viral protein and infectious virus production.

### AO13 reduced the levels of infectious virus produced from a single-cycle virus production assay.

**FIG 3:**
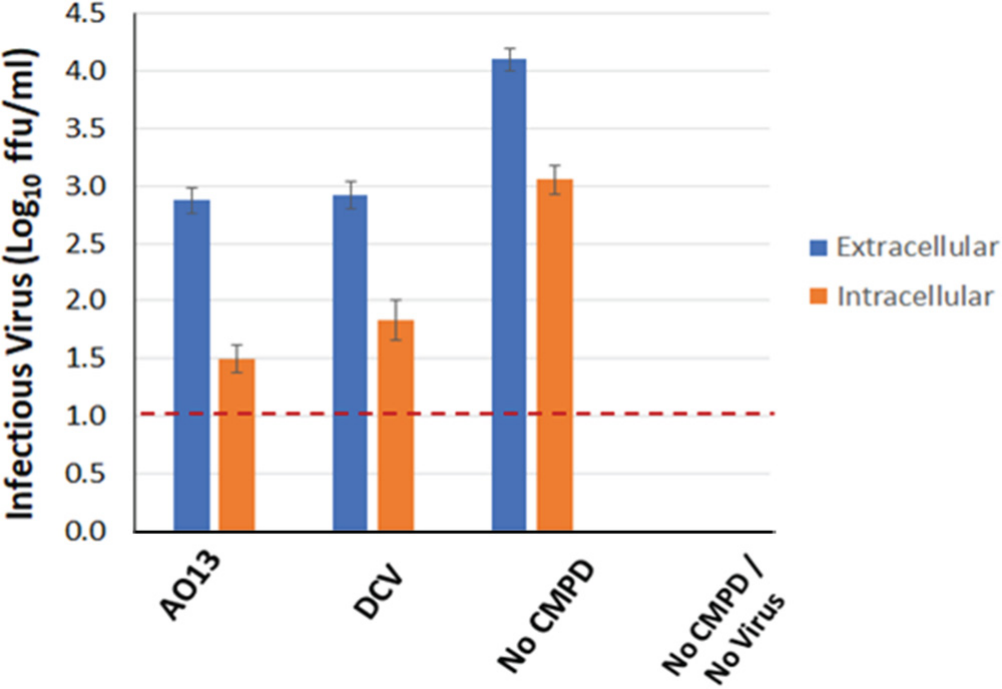
The effect of AO13 and DCV on virus release in cell culture was analyzed by comparing viral titers in extracellular (blue bars) versus intracellular (orange bars) in S29 cells. Concentrations of 10 μM AO13 or 100 pM DCV were used for all experiments. Virus titrations were performed in triplicate and results shown are representative of two independent experiments. CMPD, compound.

Based on the reduction of extracellular viral RNA and intracellular core protein observed above, we used a single-cycle virus production assay to specifically examine the effects of the compound on RNA replication and postreplication steps of the viral life cycle. We possess a subclone of Huh-7 cells (S29 cells) that can be transfected with HCV RNA to produce infectious virus, but since these cells are 1,000-fold less permissive for HCV infection than Huh-7.5 cells due to very low levels of CD81, they provide a single readout of virus production in the absence of entry/fusion/uncoating steps (25). Therefore, any effects observed in this assay are due to inhibition of viral RNA replication or subsequent steps in protein processing or virus release. S29 cells were seeded at 1 × 10^6^ cells per 10-cm dish and transfected the following day with *in vitro*-transcribed JFH1_T_ RNA. At 3 h posttransfection, the cells were treated with 10 μM AO13 or 100 pM DCV and were cultured for 2 days. When the levels of infectious virus produced from these treated cultures were measured, we found that AO13 and DCV each caused a 1-log reduction in infectious virus produced (Fig. 3, blue bars). Note that the titers are typically lower overall in S29 cells because of the single-cycle nature of virus production in these cells. This finding is consistent with the effects observed in the other experiments described above. Based on this result, we concluded that AO13, like DCV, was acting at or after the stage of HCV RNA replication.

### AO13 does not affect virus release.

To determine whether AO13 directly affects infectious virus release, we also measured the levels of intracellular infectious virus present in the transfected S29 cells treated with AO13 or DCV. To do this, culture fluids were collected (extracellular virus) and transfected cells were trypsinized and pelleted by centrifugation at 400 × *g* and then resuspended in 1 ml of complete Dulbecco modified Eagle medium (DMEM). The cells were then subjected to three rounds of freeze-thaw cycles in a dry ice-methanol bath. Cellular debris was pelleted, and the levels of infectious virus were measured in the cytosolic lysates (intracellular virus). In this assay, a difference in the ratios of extracellular to intracellular infectious virus indicates a defect in virus release from the cells. Consistent with the results above, we observed an overall reduction in both extracellular and intracellular pools of virus when transfected cells were treated with either AO13 or DCV (Fig. 3, blue versus orange bars). However, the relative ratios of extracellular versus intracellular infectious virus in the context of both AO13 and DCV treatment were similar to that of the No CMPD control. These results clearly show that AO13, like DCV, affects a life cycle step upstream of virus release.

### Docking studies suggested the HCV NS5B polymerase as the potential target for AO13.

**FIG 4:**
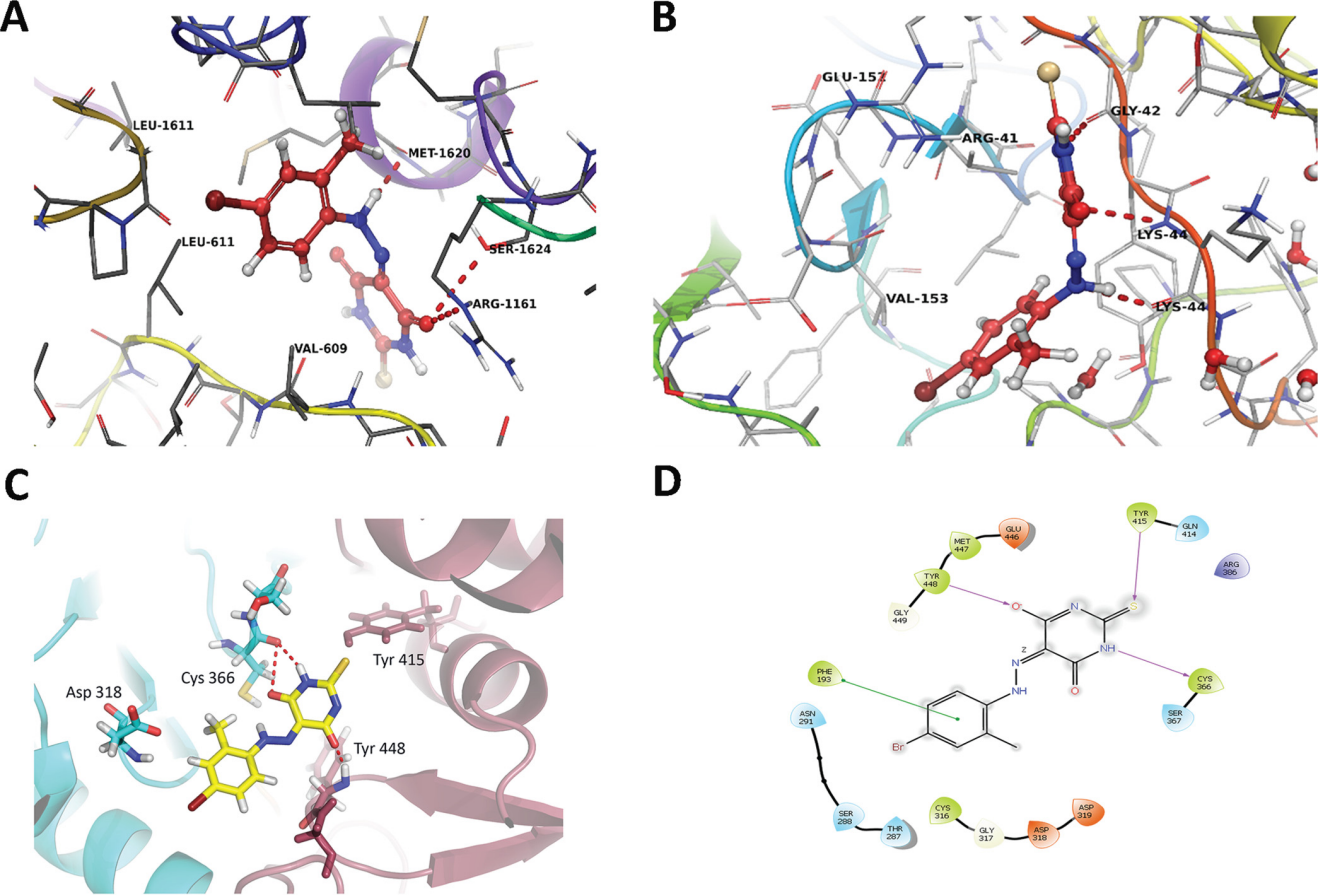
Molecular docking of AO13 to HCV viral proteins. (A) Interactions of AO13 with NS3/4A protease and NS5A (molecular docking). (A) AO13 was docked into NS3/4A (PDB 1CU1). The N of NH (diazo-phenyl) interacted with Met 1620 and O-2 of the thioxodihydropyrimidine interacted with Ser 1624 and Arg 1161 away from the active site. (B) Docking of AO13 into NS5A (PDB 3FQQ), and three hydrogen bonds are shown from that interaction. N-1 of thioxodihydropyrimidine formed a hydrogen bond with Gly 42, and O-1 formed a hydrogen bond with the amide backbone of Lys 44. Another hydrogen bond was shown by the interaction of the N of NH (diazo-phenyl) with the carbonyl group of Lys 44. (C) Interactions of AO13 at the active site of HCV NS5B polymerase. AO13 was docked into NS5B polymerase of JFH1_T_ (PDB 4AEP). N-1 of the thioxodihydropyrimidine interacted with Cys 366 and O-2 interacted with Tyr 448. Phe 193 is not shown for figure clarity. (D) Ligand interactions represented in a two-dimensional diagram.

The results described above pinpoint the activity of AO13 to some aspect of viral RNA replication or polyprotein production or processing. Therefore, we used molecular docking studies to attempt to identify the possible binding site of our compound and to reveal a potential mechanism of action. We used the Glide docking protocol at standard precision as our docking protocol. Investigating the binding mode of AO13 to NS3/4A and NS5A protein showed that the compound did not fit into the active binding pockets of either protein. The binding interactions of AO13 with NS3/4A protease (PDB 1CU1) showed three hydrogen bonds to Met 1620, Arg 1161, and Ser 1624, which are outside the reported active site of HCV protease (Fig. 4A) (26). Docking of AO13 into the NS5A X-ray structure (PDB 3FQQ) showed that the compound could potentially form three hydrogen bonds to Lys 44 and Gly 42, away from the Tyr 93 that is the location of known NS5A inhibitor resistance mutations (27). Furthermore, the docked molecule was not in direct contact with this important residue **(**Fig. 4B).

Interestingly, AO13 showed two potential hydrogen bonds at Tyr 448 and Cys 366, which were reported to be the most important residues in the active site cavity within the palm allosteric site of the NS5B polymerase (28, 29). AO13 fitted well into the allosteric binding pocket with another hydrogen bond between the sulfur atom and Tyr 415. Another noncovalent interaction was revealed from this binding pose in which the phenyl ring of the ligand formed a π-π stacking with Phe 193, which would contribute compound stability within the active site (Fig. 4C and D). Taken together, these docking analyses suggested that the possible target of AO13 could be the HCV NS5B polymerase.

### Molecular dynamics simulations suggested a stable binding of AO13 to NS5B.

**FIG 5:**
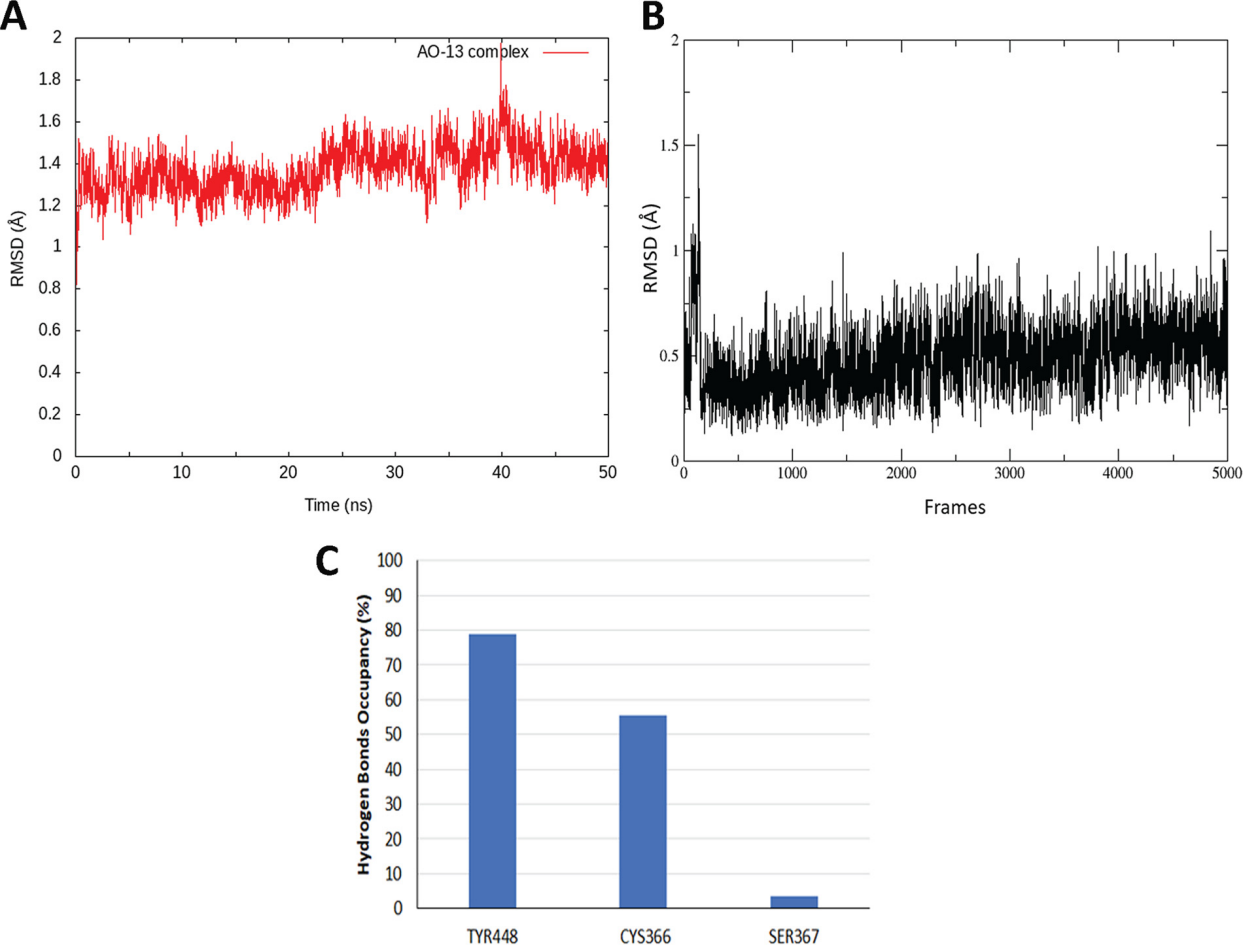
Molecular dynamics analysis of AO-13 in complex with NS5B. (A) RMSD trend of the complex backbone for AO13-NS5B complex showing stability of compound AO13 during the simulation time with an RMSD value of 1.9 Å. (B) RMSD of the ligand (AO-13) extracted from the MD trajectory showing a stable and little deviation of AO-13 during the 50-ns simulation with an RMSD of 0.48 Å. (C) Occupancy of the hydrogen bonds in the 50-ns simulation of compound AO13 in complex with HCV NS5B. Compound AO13 formed 78% occupancy with Tyr 448 and 55% occupancy with Cys 366 during the 50-ns production simulation. A hydrogen bond is assumed to exist if the donor-acceptor distance is smaller than 3.5 Å.

The main purpose of the molecular dynamics (MD) simulation studies was to investigate the conformational changes of the ligand in relation to the binding site that provides insight into the binding stability. MD revealed that AO13 could efficiently bind to the palm allosteric site which reveals the potential activity of this compound. To evaluate the stability of the NS5B protein complex during MD simulations, the root mean square deviation (RMSD) value was calculated over the 50-ns simulation time. The trajectories indicated that compound AO13 was stable in the palm site during the simulation with a mean RMSD value of 1.9 Å (Fig. 5A) The compound showed a very stable complexation with little deviation from its original coordinates with an RMSD value of 0.48 Å (Fig. 5B). Hydrogen bond occupancy analysis showed the interaction between AO13 and Tyr 448 of NS5B has an estimated occupancy of 78% during the 50-ns simulations. Moreover, the estimated occupancy was 55% between AO13 and Cys 366 (Fig. 5C). These results supported the docking results and proved the stability of the compound docking to HCV NS5B.

### AO13 has a minimal effect on the levels of intracellular HCV RNA.

**FIG 6:**
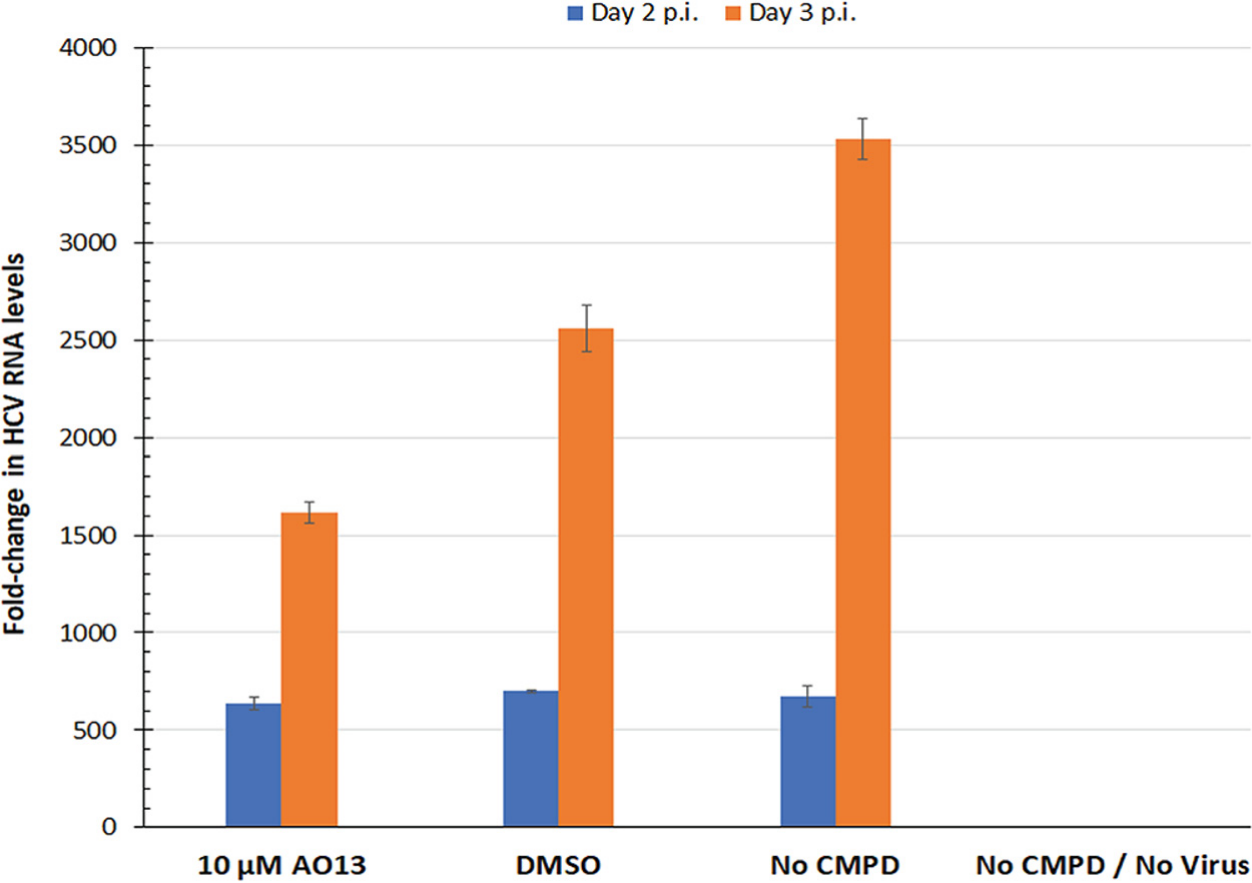
Effect of AO13 on intracellular HCV RNA levels. Huh-7.5 cells were infected at an MOI of 0.1 with JFH1_T_. Cells were treated with no compound, DMSO at a 1:1,000 dilution in complete medium, or 10 μM AO13 in complete medium. RT-qPCR was performed on cellular RNA extracts from cells harvested at day 2 (blue bars) and day 3 (orange bars) postinfection (p.i.). Cellular HCV RNA levels were quantified in triplicate relative to GAPDH RNA levels. The fold change in HCV RNA levels was calculated relative to day 3 uninfected cells. The results shown are representative of three independent experiments. Error bars represent ± the standard error. Statistical analysis was performed using analysis of variance within Microsoft Excel (****, *P* ≤ 0.0001).

To test whether the mechanism of action of AO13 was inhibition of RNA replication, we performed semiquantitative real-time RT-PCR on the levels of intracellular HCV RNA in the presence versus absence of AO13 treatment. One million Huh-7.5 cells were seeded in 10-cm dishes 24 h before inoculation with HCV at an MOI of 0.1. Infections were treated with 10 μM, a concentration equivalent to that for DMSO, or left untreated. Cells were harvested on days 2 and 3 p.i., and total RNA was extracted and subjected to real-time RT-PCR using TaqMan primer/probe sets specific for HCV and GAPDH (glyceraldehyde-3-phosphate dehydrogenase). The levels of HCV RNA in each sample were then expressed relative to GAPDH expression, and the effect of AO13 on RNA replication was assessed based on the changes in HCV RNA levels compared to that of GAPDH, expressed as ΔΔ*C_T_*. When we compared fold change (2^ΔΔ*C_T_*) among the various treatments (Fig. 6), we observed an ∼2-fold reduction in relative HCV RNA levels in the AO13-treated versus untreated infected cells. Based on these results, we conclude that if AO13 is directing acting on any component of the HCV replicase, the impact on genome replication is minimal.

## DISCUSSION

In this study, we aimed to test whether a novel synthetic compound could affect HCV production using an HCV cell culture system (HCVcc). This novel substituted phenylazo-thioxodihydropyrimidine compound (AO13) was evaluated against the commercially available NS5A inhibitor, daclatasvir (DCV) in the context of a cell culture-adapted strain of HCV JFH-1 (JFH1_T_) in human hepatoma-derived Huh-7.5 and related S29 cells. We found that our compound AO13 inhibited virus spread and reduced the levels of infectious virus produced in cell culture (Fig. 1). The extent of inhibition by AO13 was relatively low, at approximately 10- to 100-fold, but was consistent across multiple methods of virological analysis. We showed by time of addition assays that the presence of the compound in the cells before or at the time of virus infection reduced the amount of infectious virus produced. Subsequent experiments aimed at identifying the mechanism of action showed reductions in HCV core protein levels in infected cells, suggesting that AO13 acts on some aspect of viral RNA replication or protein production or processing (Fig. 2). Comparison of extracellular versus intracellular levels of infectious virus in cells treated with AO13 demonstrated that the compound does not affect virus release from cells (Fig. 3).

The main question remaining at this point is with regard to the specific mechanisms of action of this compound. Initial results suggested that the compound acts on an early stage of the virus life cycle since pretreatment of target cells showed greater inhibition than did addition of compound after virus inoculation (Fig. 2). However, subsequent results suggested that the compound affected virus kinetics at or after RNA replication. If the compound is indeed acting on RNA replication, then presumably the effect we observed upon pretreatment was due to the fact that the compound might have already been present in the cell at the time of initiation of RNA replication, which would be consistent with the observed decrease in HCV core protein levels in infected cells (Fig. 2). When DCV was included for comparison purposes, similar results were always observed with AO13 as were with DCV, again implying that AO13 also acts during viral genome replication. It is important to note here that NS5A is also thought to play a role in virus assembly (30). Thus, it is also possible that AO13 affects virus assembly and that the reduced levels of core protein we observed could have been an indirect effect resulting from an overall reduction in virus spread. The most conclusive data in this study were generated using our single-cycle virus production assay, in which only life cycle steps from RNA replication to virus release are recapitulated. In this assay, we observed a clear and consistent reduction in virus output. These data paired with the demonstrated lack of effect on virus release conclusively places the mechanism of action between RNA replication and virus assembly. To further address the mechanism of action of this compound, follow-up studies are currently planned aimed at analyzing the potential for AO13 to affect virus assembly. We also plan to attempt to generate phenotypic resistance against this compound, which would pinpoint where and on which HCV protein AO13 might be binding.

To shed additional light onto the potential mechanism of action of AO13, we used a molecular docking approach to reveal the possible target site of the compound on known HCV drug targets, i.e., NS3/4A protease, NS5A, and NS5B polymerase. Initially, we explored the possibility that AO13 is acting on the NS3/4A protease. The results from the docking into NS3/4A revealed that the compound could potentially attach to the protein by three hydrogen bonds at Met 1620, Arg 1161, and Ser 1624, but since these are distant from the active site and the reported residues involved in protease inhibitor drug resistance, it is unlikely that AO13 binds to NS3/4A (31).

Since AO13 is structurally different from most NS5A inhibitors and lacks the central biphenyl rings that typically fit into the hydrophobic cavity within NS5A, we did not expect the compound to show efficient docking results with this protein. To confirm our expectations and to exclude this possibility, we performed docking analyses between AO13 and NS5A and found three potential hydrogen bonds to Lys 44 and Gly 42, which are distal to common regions of NS5A reported to be the targets of NS5A inhibitors (27, 32).

The structure of the HCV NS5B polymerase revealed that the catalytic domain has a right-hand configuration with fingers, palm, and thumb domains typical of polymerase enzymes. The discovery and development of HCV NS5B polymerase active site-directed nucleoside and nucleotide inhibitors and their prodrugs, as well as nonnucleoside inhibitors that bind in the three allosteric sites (thumb I, thumb II, and palm site), have been reviewed extensively (33, 34). Based on our virological data, docking analyses and MD simulations, we proposed that the mechanism of action of AO13 might be inhibition of the NS5B polymerase. Docking studies performed on HCV JFH1 NS5B (PDB 4AEP) revealed that AO13 could bind at the base of the active site cavity within the palm allosteric site. The O-4 of the thioxodihydropyrimidine ring potentially forms a hydrogen bond with backbone amide nitrogen of Tyr 448, and the N1 appears to be able to form another hydrogen bond with Cys 366. Moreover, the sulfur atom may form a hydrogen bond with Tyr 415. Investigating the binding mode of AO13 indicated a π-π stacking with Phe 193, which would enhance the stabilization of the compound in the allosteric site. Binding of AO13 to important residues within the palm I region, i.e., Tyr 448, Cys 366, and Tyr 415, predicts that the compound could potentially act as an HCV NS5B polymerase inhibitor (28, 29). It is important to note here that these residues are conserved within multiple crystal structures of NS5B (35, 36). The analysis of the potential binding mode of compound AO13 at the palm allosteric site of NS5B and the MD simulations along with the antiviral results suggested that this scaffold to which AO13 belongs could be a lead for further structural modifications to develop and optimize new class of polymerase inhibitors.

In an effort to confirm a direct effect of AO13 on NS5B or some component of the HCV replication complex, we tested whether AO13 treatment affected the levels of intracellular HCV RNA. We observed a consistent, but minimal ∼2-fold reduction in the levels of HCV RNA relative to GAPDH when we performed semiquantitative real-time RT-PCR on HCV-infected cells in the presence or absence of compound (Fig. 6). Initially, we interpreted this mild reduction to mean that AO13 does not inhibit any component of the HCV replication complex. However, given that the effect of AO13 on infectious virus production was at maximum a 10- to 100-fold reduction, and assuming the relationship between intracellular genome copies and infectious progeny is not likely to be a linear one, it is possible that a 2-fold reduction in genome replication efficiency could translate to a 10- to 100-fold reduction in infectious particle production. However, it is not possible at this time, based on the data at hand, to confidently conclude that AO13 is acting directly on NS5B or any other replicase component.

Significant progress has been made in recent years regarding drug development and treatment for HCV infection. However, access to care remains to be a barrier to treatment and cure rates are lower for some HCV genotypes than others (37). This, along with what are still extremely high costs for therapy, presents a continued need for new antiviral agents and new classes of antiviral agents, especially if we think outside of HCV and consider the threat of emerging and reemerging virus infections (38). Hence, we reported here the discovery and evaluation of a new compound exhibiting potential anti-HCV activity. In all likelihood, we have identified a novel compound that acts on HCV RNA replication, and we have many of those already in the clinic. However, further investigation into the specific mechanism of action of AO13 could theoretically identify a novel class of antivirals that might be useful in the treatment of HCV, or more importantly, against another flavivirus or nonflavivirus for which we currently do not have effective therapies. In this regard, AO13 could represent a starting point for new potential antiviral agents.

## MATERIALS AND METHODS

### Compounds.

Compound AO13 [5-(2-(4-bromo-2-methylphenyl)hydrazono)-2-thioxodihydropyrimidine-4,6(1*H*,5*H*)-dione] (Fig. 1) was synthesized according to the synthetic route reported previously and showed >95% purity (39). Daclatasvir (DCV) was purchased from Sigma.

### Cells.

Infection and transfection experiments were performed in Huh-7.5 (25) and S29 (23) cells at 37°C in the presence of 5% CO_2_. All cells were propagated in complete medium (CM) consisting of DMEM (Invitrogen) supplemented with 10% fetal bovine serum (Invitrogen) and 1% penicillin-streptomycin (Sigma).

### Virus stocks.

JFH1_T_, a genotype 2a cell culture-adapted strain of JFH-1, harbors three adaptive mutations within the E2, p7, and NS2 proteins (23, 24). To generate virus stocks, six 10-cm culture dishes were seeded with 1 × 10^6^ Huh-7-5 cells and cultured overnight. On the next day, a previously titered virus stock was inoculated onto the virus-naive Huh-7.5 cells for 3 h at a multiplicity of infection (MOI) of 0.1. After inoculation, culture medium was replaced with fresh complete medium, and cells were incubated for 3 days. Culture fluids were then harvested and pooled to make a bulk stock, and then a portion was inoculated onto 8-well immunofluorescence chamber slides for titer determination.

### RNA transfection.

An amount of 1 μg of linearized JFH1_T_ DNA plasmid was transcribed *in vitro* with the T7 RiboMAX Express Large kit (Promega) according to the supplied protocol. One million S29 cells were seeded in 10-cm culture dishes and allowed to adhere overnight. The following day, cells were washed twice with serum-free DMEM and then transfected for 3 h with 4 μl of the 10-μl RNA preparation using DMRIE-C transfection reagent (Invitrogen). Transfected cells were then washed once with complete medium and cultured at 37°C. For time course experiments, AO13 was added to culture fluids at indicated time points. At 48 h posttransfection, culture fluids were harvested and cells were trypsinized. Culture fluids and cell pellets were stored at −80°C for subsequent virus titrations and RT-PCR analysis.

### Virus infection for determination of the antiviral effect.

One million Huh-7.5 cells were seeded on 10-cm cell culture dishes 24 h prior to infection. The following day, the medium was removed, and virus was inoculated onto the cells for 3 h at an MOI of 0.1. After 3 h, infection medium was replaced with fresh medium, compounds were added, and cells were cultured for 2 days. Supernatants were then harvested and stored at −80°C for subsequent titrations.

### Virus titration.

Virus titers were determined by limiting dilution focus-forming unit (FFU) assay as described previously (40). Briefly, 8-well chamber slides were seeded with 50,000 Huh-7.5 cells per well 24 h prior to infection. Virus-containing culture fluids were serially diluted 10-fold with complete medium, and 100 μl of each dilution was added in triplicate to respective wells in the chamber slide. Inocula were removed 3 h postinfection, and cells were washed with complete medium. At 48 h postinfection, cells were fixed and visualized by indirect immunofluorescence for HCV core protein. Virus titers were determined by counting the number of foci in the highest dilution positive for core protein and expressed as the number of FFU/ml of supernatant, where a focus was defined as a cluster of three or more infected cells.

### Titration of intracellular infectious virus.

Cells were trypsinized at 48 h postinfection, pelleted by centrifugation for 5 min at 400 × *g*, and resuspended in 1 ml of complete medium. The resuspended cells were then lysed by three freeze-thaw cycles of (3-min freeze/3-min thaw) in a dry ice-methanol bath and pelleted by centrifugation for 10 min at 1,500 × *g*. Virus titers were determined on clarified supernatants as described above.

### Indirect immunofluorescence.

Culture medium was removed from the wells of the 8-well chamber slides, and cells were washed by immersing the slide in phosphate-buffered saline (PBS [pH 7.4]) for 2 min. Cells were fixed by immersing the slides in acetone for 1.5 min. To visualize infected cells, wells were covered with mouse monoclonal anti-HCV core antibody (B2; Anogen) diluted 1:200 in 5% bovine serum albumin in PBS for 20 min at room temperature. Slides were washed in PBS for 5 min and then incubated for 20 min with the secondary antibody (goat anti-mouse Alexa Fluor 488; Invitrogen) diluted 1:100 in PBS. Slides were then washed and mounted with Vectashield Hard Set mounting medium containing (4′,6′-diamidino-2-phenylindole; Vector Laboratories). The slides were examined at ×10 and ×20 magnifications on a Zeiss Axio Imager.M2 immunofluorescence microscope.

### Real-time RT-PCR quantitative analysis.

Viral RNA was extracted from infection culture medium using a QIAamp Viral RNA minikit (Qiagen) as per the manufacturer’s recommendations, and quantitative RT-PCR was performed as previously described (41). Primers and probe were selected from a highly conserved region of the 5′ untranslated region (5′ UTR) (42). RT-PCRs were performed with the TaqMan one-step RT-PCR Master Mix Reagents (Applied Biosystems). *In vitro*-transcribed JFH1_T_ RNA was quantified by NanoDrop and genome copy numbers estimated based on base composition and molecular weight. From this quantified preparation, a set of HCV RNA standards of known genome copy number was prepared and used to determine HCV RNA copy numbers in samples derived from virus culture. Samples were tested in duplicate in every run, data analyses were carried out using ABI’s SDS version 2.2, and the numbers generated were converted to copies/ml.

To analyze intracellular levels of viral RNA, one million Huh-7.5 cells were plated in 10-cm cell culture dishes 24 h prior to infection and/or AO13 treatment. On the day of infection, the cell medium volume per plate was reduced to 1.5 ml. Cells were inoculated for 3 h at an MOI of 0.1 with JFH1_T_. After 3 h, infection medium was replaced with fresh medium, compounds (1:1,000 DMSO or 10 μM AO13 in complete medium) were added, and cells were cultured for 2 or 3 days. Cells were harvested using 0.25% trypsin-EDTA (catalog no. 25200072; Gibco) and rinsed twice by resuspending in 1× PBS and pelleting by centrifugation. Viral RNA was extracted from cells using the QuickExtract RNA extraction kit (QER090150; Lucigen) as per the manufacturer’s recommendations. Primers and probes for HCV were selected from a highly conserved region of the 5′ UTR (Pa03453408_s1; Thermo Fisher Scientific) (42). Primers for GAPDH were designed from a singular exon (forward primer sequence GGTGGTCTCCTCTGACTTCAACA; Invitrogen). GAPDH primer/probe mix was prepared using the TaqMan QSY probe (catalog no. 4482777; Applied Biosystems). RT-qPCRs were performed in triplicate with the TaqMan Fast Virus 1-Step Master Mix (catalog no. 4444432; Applied Biosystems) according to the manufacturer’s instructions. Samples were run using the Applied Biosystems 7500 Fast real-time PCR system. Applied Biosystems 7500 Software version 2.0.5 generated *C_T_* and Δ*C_T_* values, which were used to calculate ΔΔ*C_T_* and fold change (2^ΔΔ*C_T_*).

### Western immunoblot analysis.

Samples were subjected to 10% sodium dodecyl sulfate-polyacrylamide gel electrophoresis (SDS-PAGE) at 100 V for 2 h. Separated proteins were then transferred to a nitrocellulose membrane (Bio-Rad) in a Trans-blot Turbo Transfer machine (Bio-Rad) for 7 min. The membrane was blocked with 5% skim milk in TBS-T (20 mM Tris, 137 mM NaCl [pH 7.3], 0.05% Tween 20) for 1 h, followed by incubation with anti-HCV core protein antibody (B2; Anogen) or GAPDH antibody (6C5; Abcam) overnight. Next day, the membrane was washed three times with TBS-T and incubated for 1 h with peroxidase-conjugated goat anti-mouse IgG secondary antibody (Santa Cruz). The membrane was washed again in TBS-T three times and developed using chemiluminescent substrate (Thermo Fisher Scientific) and a luminescent image analyzer (Image Quant LAS4000) for detection of specific bands.

### Molecular docking.

The molecular structure of AO13 was built using ChemBioDraw Ultra version 14.0, and its energy was minimized using the MMFF94x force field with ChemBio3D Ultra to produce the lowest energy conformer. All molecular docking experiments were performed using the Schrödinger Small Molecule Discovery Suite. The Protein Preparation Wizard module was used to add hydrogen atoms, minimize energy, and create appropriate protonation states of amino acid side chains. The docking algorithm Glide was used to perform all molecular docking studies (43). The PDB X-ray structures (PDB 1CU1, 3FQQ, and 4AEP) for NS3/4A, NS5A, and NS5B, respectively, were imported into Maestro and processed using Protein Preparation Wizard. The protein structure was then subjected to three stages of energy minimization, all of which utilized the OPLS3 force-field (16, 44). A receptor grid file was generated based on the prepared protein active site(s) and the SiteMap utility in Schrodinger Suite (45). The Glide docking algorithm at standard precision (SP) was carried out with the receptor grid file and the prepared ligand. Hydrogen bonding, Van der Waals, π-π interactions of AO13 with amino acid residues, and the ligand poses were calculated and analyzed.

### Molecular dynamics simulations.

The force field parameters for selected ligands have been obtained using the GAFF force field (46) and Antechamber (47). The AM1-BCC charges were assigned for the ligands (48), and the ff09SB parameters were assigned for the protein. The complexes have been neutralized, solvated in a NaCl concentration of 0.15 M with tleap using the same process as described above. The simulations have been performed in pmemd.cuda: an initial minimization step was performed in order to relax the water and ionic positions. The whole system was then minimized and heated gradually up to 310K in 100 ps using Langevin dynamics. During the heating process, we restrained the backbone of the protein and the heavy atoms of the ligand; a time step of 0.5 fs and periodic volume conditions have been employed during this phase. The time step has been set to 2 fs, periodic pressure conditions (1 atm) have been imposed and the restraints have been gradually released in four phases of 50 ps each. The simulations were then continued for 50 ns. During the MD simulations, the equations of motion were integrated using a 2-fs time step and the atomic coordinates were saved to the trajectory producing 5,000 frames. Visualization of protein-ligand complexes and MD trajectory analysis were carried out using CPPTRAJ (49) and VMD.
